# A comprehensive review of the epidemiology and disease burden of Influenza B in 9 European countries

**DOI:** 10.1080/21645515.2015.1111494

**Published:** 2016-02-18

**Authors:** Monica Tafalla, Marleen Buijssen, Régine Geets, Marije Vonk Noordegraaf-Schouten

**Affiliations:** a GSK Vaccines, Wavre, Belgium; b Pallas, AE Rotterdam, the Netherlands

**Keywords:** co-circulation, Europe, influenza B, influenza vaccine, lineages, quadrivalent, trivalent, vaccine mismatch

## Abstract

This review was undertaken to consolidate information on the epidemiology and burden of influenza B, as well as the circulation patterns of influenza B lineage in 9 European countries. Following a comprehensive search of peer-reviewed and gray literature sources, we found that published data on influenza B epidemiology and burden are scarce. Surveillance data show frequent co-circulation of both influenza B lineages during influenza seasons, but little is known about its impact, especially in adults and the clinical burden of influenza B remains unknown. Mismatch between the circulating influenza B lineage and vaccine recommendations has been seen in at least one influenza season in every country. Such observations could impact the effectiveness of seasonal influenza vaccination programs using trivalent vaccines, which contain only one influenza B lineage (B/Yamagata or B/Victoria) and highlight the need for local studies to better understand the epidemiology and burden of influenza B in these countries.

## Introduction

Annual seasonal influenza epidemics, caused by influenza virus types A and B, are associated with considerable health and economic consequences worldwide,^1^ and are estimated to cause about 3–5 million cases of severe illness, and about 250–500,000 deaths each year. The estimated annual attack rates range from 5–10% in adults to 20–30% in children.^2^ The burden of seasonal influenza in Europe is therefore large and associated with high cumulative social and economic burden manifesting, not only as direct medical costs, but also as reduced quality of life and lost work productivity.^3^

Although there is general belief that influenza disease due to type B is milder than type A, data show that infections with influenza A and B are clinically indistinguishable.^4,5^ Individuals of all ages can be affected, but the incidence of influenza complications is generally higher in younger children and the elderly. ^6^ Notably, higher rates of disease-associated complications and deaths are seen in the elderly due to the frequent presence of underlying chronic health conditions.^6^

Several disease management strategies including vaccination and antiviral treatment are available to cope with seasonal influenza epidemics.^7^ Vaccination is considered the most effective option for preventing influenza related-illnesses and complications.^7^ The most commonly used vaccine in influenza immunization programs worldwide is the trivalent formulation which comprises virus types representing 3 influenza strains – 2 influenza A strains (A/H1N1 and A/H3N2), and one influenza B lineage (B/Yamagata or B/Victoria). As influenza viruses undergo frequent changes in their surface antigens, new influenza vaccines are designed annually to match the circulating lineage expected for the next influenza season.^8,9^

Epidemiological surveillance data show widespread co-circulation of these 2 B lineages within the same influenza season since 2000.^10,11^ As one of these lineages is not covered by the annual vaccine, and due to limited cross-protection conferred between the 2 antigen-distinct influenza B lineages, a vaccine lineage mismatch with circulating lineages could result in less effective vaccination programs and additional disease-related burden.^12-17^

Quadrivalent influenza vaccines, which contain 2 influenza A (A/H1N1 and A/H3N2) and 2 influenza B lineages (B/Victoria and B/Yamagata), have been developed as an alternative, to avoid poor antigenic match and improve protection by offering broader protection against influenza B.^7,18^ Accordingly, WHO (World Health Organization) updated its vaccine recommendations for the Northern Hemisphere during the 2012–2013 influenza season to include a quadrivalent vaccine formulation for the prevention of seasonal influenza.^19^ In line with this recommendation, it is expected that quadrivalent vaccines could prove beneficial over trivalent vaccines in the control of seasonal influenza.

Due to competing health priorities, accurate and consistent epidemiological evidence is needed to support appropriate and relevant vaccine-specific recommendations. However, there is a lack of evidence on the epidemiology and pattern of circulation of influenza B in some European countries. The aim of this literature review was therefore to examine the epidemiology, disease burden, seasonality and strain circulation patterns of influenza B in the overall population of 9 small to medium-sized European countries (Switzerland, Austria, Belgium, Luxembourg, Finland, Greece, Czech Republic, Slovakia and Estonia).

### Comprehensive review of peer-reviewed literature

**Figure 1.**
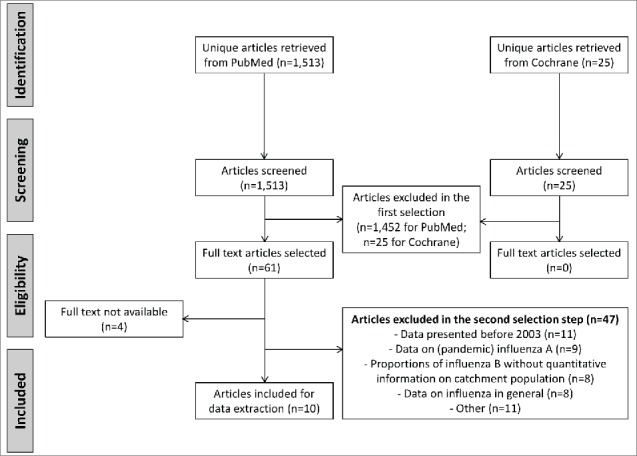
PubMed selection procedure and number of included articles.

The literature searches identified 1,513 hits in PubMed and 25 in the Cochrane library, from which 10 peer-reviewed articles were included in the final review (Switzerland [n = 1]; Austria [n = 1]; Belgium [n = 1]; Luxembourg [n = 0]; Finland [n = 2]; Greece [n = 3]; Czech Republic [n = 1]; Slovakia [n = 1]; Estonia [n = 0]). All of the articles provided information on disease epidemiology, 6 had information on disease burden (General Practitioner [GP] and Emergency Room [ER] visits, hospitalization rates, mortality, complications and antibiotic use) and 3 had information on circulating strains. All articles had been found in PubMed; no articles were retrieved from the Cochrane library or manual literature search (Fig. 1).

#### Epidemiology of influenza B infection

No data on the incidence of the 2 influenza B lineages were identified in the review. Some information was available on the proportions of influenza B lineage among laboratory-confirmed influenza cases for all countries except Luxembourg and Estonia (Table 1). The majority of studies reporting the proportion of influenza B lineage circulation considered only pediatric populations. Data on the age distribution of influenza B cases were scarce.^20-23^

**Table 1.** Summary of influenza B burden of disease from the literature review.

			Proportions, n (%)			
Country	Season	Population	Laboratory-confirmed influenza	Influenza B	Influenza A	Comments
Switzerland^21^	2001–2003	Pediatric patients hospitalized with respiratory symptoms during the cold season (n = 338)				—
		2001–2002 (n = 112)	27 (24.1%)	3 (11.1%)	24 (88.9%)	
		2002–2003 (n = 226)	33 (14.6%)	12 (36.4%)	21 (63.6%)	
Austria^24^	1999–2010	Swabs collected from a subset of patients from sentinel surveillance throughout Austria, and from hospitals, pediatricians and unaffiliated physicians (n = 14,539)				—
		1999–2000 (n = 319)	115 (36.1%)	0 (0.0%)	115 (100%)	
		2000–2001 (n = 288)	122 (42.4%)	12 (9.8%)	110 (90.2%)	
		2001–2002 (n = 449)	205 (45.7%)	129 (62.9%)	76 (37.1%)	
		2002–2003 (n = 874)	510 (58.4%)	45 (8.8%)	465 (91.2%)	
		2003–2004 (n = 843)	333 (39.5%)	1 (0.8%)	332 (99.2%)	
		2004–2005 (n = 1,087)	440 (40.5%)	171 (38.9%)	269 (61.1%)	
		2005–2006 (n = 859)	245 (28.5%)	67 (27.3%)	178 (72.7%)	
		2006–2007 (n = 1,827)	430 (23.5%)	4 (0.9%)	426 (99.1%)	
		2007–2008 (n = 1,533)	341 (22.2%)	53 (15.5%)	288 (84.5%)	
		2008–2009 (n = 1,513)	511 (33.8%)	88 (17.2%)	423 (82.8%)	
		2009–2010 (n = 4,974)	1,952 (39.5%)	2 (0.1%)	1,950 (99.9%)	
Belgium^20^	2009–2010	PediSurv network: Patients aged <5 years presenting with ILI (n=139).	28 (20.1%)	1 (3.6%)	27 (96.4%)	—
		Sentinel surveillance network: Patients with ILI (n = 810)	426 (52.6%)	3 (0.7%)	423 (99.3%)	
Finland^22, 27^	July 1998-June 2004 ^22^	Children aged ≤16 years who were hospitalized with laboratory-confirmed influenza (n = 401)	–	70 (17.5%)	330 (82.3%)	1 (0.2%) child had a combined influenza A and B infection
	Nov 2002-April 2003 ^27^	Patients in the Finnish Defence Forces with an upper respiratory infection (n = 302)	135 (34.7%)	109 (80.7%)	26 (19.3%)	—
Greece^23, 25, 26^	2009–2012 ^25^	Pediatric population of Southern Greece with ILI (n = 7,357)	2,656 (36.1%)	87 (3.3%)	2,569 (96.7%)	—
		2009–2010 (n = 5,137)	1,664 (32.2%)	2 (0.1%)	1,662 (99.9%)	
		2010–2011 (n = 1,870)	848 (45.3%)	14 (1.7%)	834 (98.3%)	
		2011–2012 (n = 314)	144 (45.9%)	71 (49.3%)	73 (50.7%)	
	2005–2008 (Winter seasons; November-May) 26	Pediatric population of Southern Greece with ILI (n = 1,272)2005–2006 (n = 392)2006–2007 (n = 377)2007–2008 (n = 503)	387 (30.4%)69 (17.6%)181 (48.0%)137 (27.2%)	Influenza virus type B was the dominant recovered type for the first season (83.6%), while subtypes A/H3N2 (78.1%) and A/H1N1 (70.8%) dominated the following 2 winter seasons, respectively	Distribution of influenza A and B was only presented in mixed infected cases (21.3%) and not for all virologically confirmed influenza cases	
	2002–2005 23	729 hospitalized children aged 6 months–13 years	161 (22.1%)	23 (14.3%)	134 (83.2%)	Remaining 4 (2.5%) cases were combined influenza A and B infections
Czech Republic^39^	2012–2013	Patients presenting with ARI or ILI (n = 2,793)	994 (35.6%)	30 (3.0%)	964 (97.0%)	—
Slovakia^40^	1993–2008	Laboratory-confirmed influenza cases (n = 1,298)	–	293 (22.6%)	1,005 (77.4%)	Surveillance was sentinel since 2002/2003; before this season only few samples were collected

ARI: acute respiratory infection; ILI: influenza-like illness.

#### Burden of disease due to influenza B

Information on the burden of disease was available for influenza B-associated hospitalization rate in Finland (n = 1)^22^ and influenza B-associated complications and antibiotic use in Switzerland (n = 1).^21^ No information was found either on influenza B-associated GP or ER visits or influenza B-associated mortality for any of the countries included in this review. However, 6 articles (Switzerland [n = 1]^21^; Austria [n = 1]^24^; Finland [n = 1]^22^; Greece [n = 3]^23,25,26^) presented combined data on the overall burden of influenza together with data on the proportion of influenza B: hospitalization rates (n = 3)^21-23^; mortality (n = 2)^24,25^; complications (n = 3)^21,23,26^; antibiotics consumption (n = 2).^21,23^ These data combined may provide a rough indication of the disease burden due to influenza B.

##### Influenza-related hospitalization and mortality

Data on overall influenza-related hospitalization and mortality combined with proportions of influenza B were limited^21-25^ and, where available, these data were primarily focused on the pediatric population. Information on influenza-related hospitalization and mortality were lacking for Belgium, Luxembourg, Czech Republic, Slovakia and Estonia.

Regarding overall influenza, in 2001–2003 the yearly population-based hospitalization rate for overall influenza in children aged 0–18 years in Switzerland was 39.5 per 100,000; the annual hospitalization rate in children below 6 years of age was high (141 per 100,000 children).^21^ Proportions of influenza B among hospitalized pediatric patients with laboratory-confirmed influenza were 11.1% in 2001–2002 and 36.4% in 2002–2003.^21^ Information on the overall influenza-related mortality rate in combination with proportion of influenza B was not available for Switzerland.

Data on overall influenza-associated hospitalization rate and associated influenza B proportions were not available for Austria. A Poisson model-based study indirectly estimated influenza-attributable deaths using weekly surveillance (included in Table 1) and daily mortality data. All-cause influenza-attributable deaths ranged from 4.4 to 50 per 100,000 per season during 1999–2009. Mortality rates were highest among the elderly aged ≥60 years. In those of ages 0–14 years and 15–24 years, influenza-associated mortality during the seasonal epidemics ranged from 0 to 2.1 and 0 to 2.7 per 100,000, respectively.^24^ The overall proportion of influenza strains is presented in Table 1. In Finland, the average annual incidence of overall influenza- and influenza B-associated hospitalization in infants below one year of age was 225 (95% Confidence Interval [CI]: 188–262) and 30 (95% CI: 17–44) per 100,000 children respectively.^22^ No data on influenza-associated mortality was identified in the review.

Overall influenza-associated hospitalization rates in the Athens area were 16.8 per 10,000 children in 2002–2003 and 13.6 per 10,000 children in 2004–2005.^23^ The proportion of influenza B among all influenza-associated hospitalizations was 14.3% for both seasons combined. Hospitalization rates were highest in children under 5 years of age and particularly among those below 3 years of age.^23^ The following mortality rates were reported for a pediatric population in southern Greece: 2.0%, 6.5% and 9.0% of influenza cases in 2009–2010, 2010–2011 and 2011–2012 seasons, respectively. The proportions of influenza B among all influenza-related hospitalizations during these seasons were 0.1%, 1.7% and 49.3%, respectively.^25^

##### Rates of influenza-related complications and antibiotic use

Three studies (Switzerland [n = 1]^21^; Greece [n = 2]^23,26^) provided some information on the frequency of complications and antibiotic use resulting from an outbreak of influenza in the region. Only one study (Switzerland)^21^ described these data specifically for influenza B; the other 2 provided information on the proportion of influenza B among the study population. Information on complications and antibiotic consumption were not available for Austria, Belgium, Luxembourg, Finland, Czech Republic, Slovakia and Estonia.

In Switzerland among the pediatric patients hospitalized with influenza B, 14.3% had febrile convulsions and 35.7% patients were prescribed antibiotics. None of the patients needed intensive care.^21^ The most frequently observed complications of overall influenza, reported in a study from Greece in 729 hospitalized children aged 6 months to 13 years, were acute otitis media (21.1%), febrile seizures (19.2%) and pneumonia (9.9%).^23^ Antibiotics were prescribed to influenza-positive patients mainly due to acute otitis media (34%) and pneumonia (16%). The proportion of influenza B in the study population was 14.3%.^23^ No statistically significant difference regarding antibiotic use was documented between the influenza-negative (343/568, 60.4%) and positive (99/161, 61.5%) groups.^23^ Data on complications in pediatric patients below 18 years in Greece during the 2005–2008 winter seasons were available from another study: 18.6%, 7.8% and 2.5% of influenza-infected patients had conjunctivitis, suffered from respiratory hindrance, and had evidence of pneumonia, respectively.^26^ Influenza virus type B was the dominant type isolated during the first season (83.6%), while subtypes A/H3N2 (78.1%) and A/H1N1 (70.8%) dominated over the following 2 winter seasons, respectively.

#### Circulation and vaccine match-mismatch of influenza B subtype lineages

Papers describing data on circulating lineages were available from only 3 studies (Austria [n = 1]^24^; Finland [n = 1]^27^; and Greece [n = 1]^25^). There was no information on circulating influenza B strains from peer-reviewed literature for Switzerland, Belgium, Luxembourg, Czech Republic, Slovakia and Estonia.

In Austria, the B/Victoria lineage was the predominant strain in the 2001–2002 season. During the other seasons between 1999–2000 and 2009–2010, influenza A strain predominated every season. No information on influenza B circulation was reported when influenza A strain predominated.^24^

In Finland information on the circulation of influenza B lineages was available only for the 2002–2003 influenza season. Both lineages of influenza B virus circulated during this season, but the B/Victoria lineage was the predominant circulating lineage (94.7%). Minor mismatch (5.3%) between vaccine lineage (B/Victoria) with the circulating B/Yamagata lineage was observed in this influenza season.^27^

In Greece, information on influenza B lineage circulation was available only for the 2011–2012 season during which both influenza B lineages co-circulated (approx. 50%); only B/Victoria lineage was included in the vaccine during this season.^25^

### Gray literature and other data sources

#### International and regional epidemiologic surveillance

##### Epidemiology of influenza B infection

**Figure 2.**
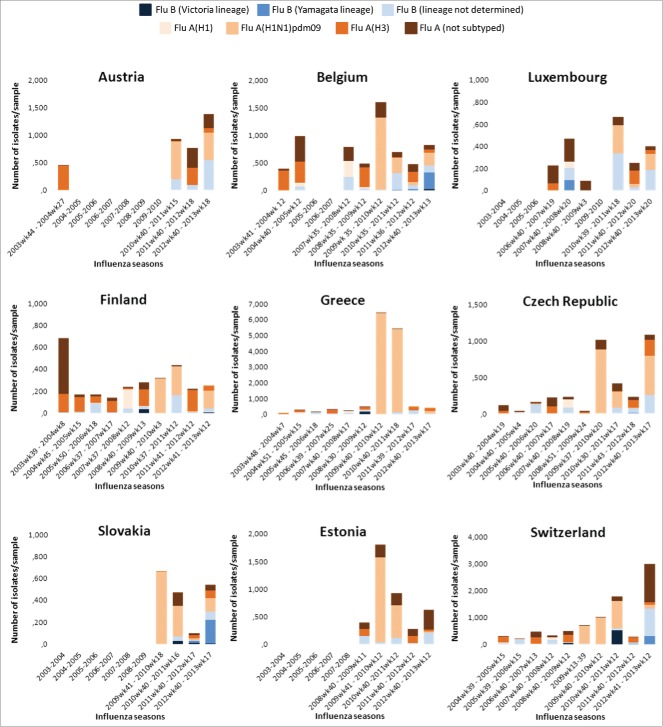
Circulation patterns of seasonal influenza viruses excluding pandemic seasons, 2003–2013* (WHO-FluNet) Flu, influenza; wk, week, *Note: Austria (Data unavailable for 2005–2009); Belgium (Data unavailable for 2006); Luxembourg (Data unavailable for 2003 and 2005); Slovakia (Data unavailable for 2005–2008); Estonia (Data unavailable for 2003–2007).

Details of the circulation of seasonal influenza strains, as obtained from WHO-FluNet database (Global Influenza Surveillance and Response System [GISRS] Laboratories) weekly reports are shown in Figure 2.

Information is lacking for some seasons and not all countries have available information on all types and subtypes. However, despite this limitation, the lack of homogeneity in seasonal strain circulation is evident.

#### Circulation and vaccine match-mismatch of influenza B subtype lineages

As no comprehensive information could be retrieved from only one source, data from WHO-FluNet and EuroFlu were combined to compile information on the circulating influenza B lineage per season (Table 2) and to assess co-circulation patterns and vaccine match-mismatch every season.

**Table 2.** Overview of circulating influenza B lineage, by country, by influenza season.

	Circulating lineage									Recommended vaccine lineage
Season	Switzerland	Austria	Belgium	Luxembourg	Finland	Greece	Czech Republic	Slovakia	Estonia	
2012–2013	Vic, Yam^*^	-nr-	Vic, Yam^*^	-nr-	Vic^*^, Yam	-nr-	-nr-	Vic, Yam^*^	-nr-	Yam
2011–2012	Vic^*^, Yam	Vic^*^, Yam	**Yam**	-nr-	-nr-	**Vic, Yam^†^**	Vic	Vic, Yam^*^	-nr-	Vic
2010–2011	Vic^*^, Yam	Vic^*^, Yam	Vic^*^, Yam	-nr-	Vic, Yam^*^	Vic, Yam^*^	Vic^*^, Yam	Vic	-nr-	Vic
2009–2010	Vic^*^, Yam	-nr-	Vic^*^, Yam	-nr-	-nr-	Vic	-nr-	-nr-	-nr-	Vic
2008–2009	Vic^*^, Yam	**Vic**	Vic^*^, Yam	-nr-	**Vic**	**Vic**	-nr-	-nr-	-nr-	Yam
2007–2008	-nr-	**Yam**	-nr-	**Yam**	-nr-	-nr-	**Yam**	-nr-	-nr-	Vic
2006–2007	-nr-	-nr-	-nr-	**Yam**	-nr-	-nr-	-nr-	-nr-	-nr-	Vic
2005–2006	-nr-	Vic^*^, Yam	Vic^*^, Yam	Yam	-nr-	-nr-	**Vic**	-nr-	-nr-	Yam
2004–2005	Vic, Yam^*^	Vic, Yam^*^	Yam	Vic^*^, Yam	-nr-	-nr-	Yam	Yam	-nr-	Yam
2003–2004	-nr-	-nr-	-nr-	-nr-	-nr-	-nr-	-nr-	-nr-	-nr-	Vic
2002–2003	Vic	-nr-	-nr-	-nr-	Vic^*^, Yam^†^	-nr-	Vic	Vic	-nr-	Vic

Note: Data are obtained from EuroFlu and FluNet, unless otherwise specified:

† Data obtained from peer-reviewed literature: Finland^27^, Greece^25^;

* Predominant lineage; Text in bold-face indicate mismatch between predominant circulating influenza B lineage and vaccine lineage; -nr-, not reported; Vic: B/Victoria lineage; Yam: B/Yamagata lineage.

From the available data, both B lineages were present at least in one season for each of the studied countries since 2002 (Table 2). Co circulation of the 2 lineages was the most frequent finding in almost all countries in the years with available information. Further, except in Estonia for which no data were available, in all countries, mismatch between the circulating influenza B lineage and vaccine recommended lineage was observed in at least one influenza season. The maximum number of mismatch years in this data series (3 of the 11 years) is seen in Austria, Belgium, Luxembourg, Finland and Greece. (Table 2). Complete vaccine mismatches (i.e. 100% circulation of the lineage other than the vaccine recommended lineage) were observed during some influenza seasons in Austria, Belgium, Luxembourg, Finland, Greece and Czech Republic.

#### National epidemiologic surveillance

No additional information was found with a search of local Ministries of Health (MoH) websites and *Google* and the retrieved information confirmed our previous findings.

## Discussion

Data on influenza B epidemiology and burden of disease are essential to understand the level of risk and protection that current trivalent vaccination can provide. In this review, we aimed to describe the available evidence on influenza B-related epidemiology and disease burden in 9 small and medium-sized European countries from the literature as well as using international and national surveillance data sources.

The first notable finding is the scarce attention that influenza B has received from researchers, as demonstrated by the low number of peer-reviewed publications covering its epidemiology. Our search identified no incidence data from these 9 countries; only strain-type distribution information was available. Burden of disease information was also scarce. Other sources of information, such as local surveillance websites or supranational organizations, which compiled the information sent by local health authorities to WHO for influenza surveillance, were very useful for our research and provided valuable information. Nevertheless, these individual sources were not sufficiently comprehensive and we needed to take information from each source to provide a bigger picture of the situation.

Reasons for this scarcity of data could include the observation that epidemiologists working in surveillance systems do not consider publishing peer-reviewed papers, as the information is disclosed in weekly reports. However this does not explain the lack of incidence data in combined peer-reviewed and other sources. As a result, clinicians cannot easily access the information. Further, assessing disease burden is a complex task, especially as in addition to the direct disease burden, an important indirect burden, due to all the medical conditions that can become complicated or even fatal because of influenza, especially in the elderly, needs to be considered. Several methods which measure the indirect influenza-attributable burden, including complex modeling exercises, are being increasingly but not routinely used. The false perception that influenza B is a mild form of influenza, can account for the lack of research, and contribute to the lack of awareness among the medical community of the true circulation, potential mismatch and disease burden.

The second important point relates to the lack of predictable patterns in strain circulation seen in these countries. Even considering neighboring countries, such as Belgium and Luxemburg in the North; Austria, Switzerland, Slovakia and the Czech Republic in Central Europe; or Finland and Estonia in the North-East, it is not possible to see any common circulation pattern. Indeed, mismatch has occurred in at least one of the 11 seasons in every country with available information (excludes Estonia) and in 3 different seasons in 5 countries; even complete vaccine mismatch was observed.

Quantitative mismatch was also highly variable, ranging from ‘very low’ when B strain proportion was very low, or ‘relevant’ during other seasons or in other countries, when there was high influenza B circulation. As there is no identifiable trend, there is no way of predicting how important it will be in future seasons.

A previous literature review of studies from 1995–2010 indicates that the burden of influenza B can be significant, regardless of age.^28^ However, studies examining hospitalization rates, ER visits and antibiotic and antiviral use associated with influenza B-attributable disease are lacking. Two previous studies reported that influenza B-related hospitalization following an ER visit is more likely in children compared to influenza A.^29,30^ Other published reports indicate that the highest burden of influenza-associated complications and death due to influenza B occurs in the elderly.^4,31,32^ Severe complications (e.g., encephalopathy, myositis) have also been linked to influenza B infection.^33,34^ It has been further shown that whereas influenza B accounts for up to 29% of respiratory influenza-attributable deaths in an average season, this could reach 51–95% of all influenza-attributable deaths in years of high influenza B circulation. ^6^ Our review of data, obtained predominantly from pediatric populations, suggested a low influenza B-specific clinical burden (ER and GP visits, hospitalization, mortality, complications and antibiotic consumption rates) in these 9 European countries. A recent published literature review by Paul Glezen *et al.* (2013) also concluded that data on influenza B are limited.^28^

Our review has some limitations, the first relating to country selection. Since our selection was based on convenience, we made no attempt to generalize our findings to any other countries or regions. However, since the value of this review relies on the lack of distribution homogeneity and is moreover based on individual country results, we believe that our results are valuable when highlighting the lack of predictable trends. Although comprehensive, we did not embark upon a systematic review as we knew beforehand that some of the information sources would not be in report form. This would have been a significant limitation for the critical appraisal phase of a systematic review, and led us to descriptively process the information. Further, in the case of influenza, as surveillance web sites at local and supranational level are an invaluable source of data on virus circulation, we felt it was important to keep these relevant sources in our review. Our results are therefore limited by the quality of the identified studies, but it should be remembered that the objective of this review was to describe all available epidemiological information. The data presented for these 9 small to medium-sized countries are considerably disparate and as such limits its impact on generalizability to the entire region of Europe. Furthermore, the results are bound by the inherent limitations of the studies included in the literature review. The comparability of data between countries are constrained by differences in individual study designs (population-based, hospital-based, etc.), outcomes, case definitions, mode of data collection (retrospective *versus* prospective) and detection methods for laboratory-confirmed influenza. In addition, drawing conclusions was hampered by gaps in laboratory surveillance, wherein some countries had no available data for many years.

In summary, our review highlights the unpredictability of influenza B lineage circulation in the region, and the mismatch between the circulating influenza B and vaccine recommended lineages in the trivalent vaccine. The findings also highlight the need for local research to better understand strain circulation and the burden of influenza, including influenza B, in the 9 European studies included in this review.

## Methods

### Comprehensive literature review

The PubMed library was used to perform a search in January 2014 for relevant literature on influenza B. Two different search strings were used in combination: (“Human influenza [MeSH] OR flu[tw] OR influenza-like illness[tw] OR flu-like illness[tw] OR influenza[tw]”) AND “(Switzerland[tw] OR Switzerland[ad] OR Swiss[tw] OR Austria*[tw] OR Austria*[ad] OR Belgi*[tw] OR Belgi*[ad] OR Luxembourg*[tw] OR Luxembourg*[ad] OR Finland[tw] OR Finland[ad] OR Finnish[tw] OR Finnish[ad] OR Greece[tw] OR Greece[ad] OR Greek[tw] OR Greek[ad] OR Czech[tw] OR Czech[ad] OR Slovak*[tw] OR Slovak*[ad] OR Estonia*[tw] OR Estonia*[ad]).”

The Cochrane database was searched by combining “influenza” with several search terms for each country: “influenza” (title, abstract, keywords) AND “Switzerland” OR “Swiss” OR “Austria*” OR “Belgi*” OR “Luxembourg*” OR “Finland” OR “Finnish” OR “Greece” OR “Greek” OR “Czech” OR “Slovak*” OR “Estonia*.” Trials, technology assessments and economic evaluations were excluded.

Limits applied in both searches included publication date (January 2003 to January 2014) and language (English, French, German and Dutch). As part of a quality control process, the first 30% of titles and abstracts were screened in duplicate by 2 independent researchers. The results were compared and discussed before the remaining references were assessed by one researcher. Subsequent to screening, the first 10% of full text articles were also critically appraised in duplicate by 2 independent researchers. The results were compared and discussed and any disagreements were adjudicated by a third researcher, when necessary.

The three step procedure for including relevant references including eligibility criteria is provided in Table 3.^35^

**Table 3.** Selection procedure.

Step 1. Screening articles by reading titles and abstracts
Inclusion criteria:
• Relevant outcome related to seasonal influenza A/H1N1, A/H3N2, B/Victoria and B/Yamagata • Conducted in, or presented data from at least one of the selected European countries• Presented data from 2003 onward
Exclusion criteria:
• Pandemic influenza • Pharmacokinetic or pharmacodynamics studies • Animal or in vitro studies • Case reports, cases series, clinical trials, or meta-analyses • Articles on pathophysiology, treatment, etiology, or diagnosis • Articles including pneumonia patients as the study population • No abstract provided (full text was checked if the title closely matched the review objective) • Fewer than 30 patients • Case studies
Step 2. Screening of full text articles selected in Step 1.
These articles were either included in the evidence tables or were excluded if the article did not contain relevant information or contained poor quality information. At this stage, critical appraisal of full text articles, using a standard set of criteria, took place. Exclusion criteria:
• A narrative review (e.g., no methods section describing how the authors collected the literature) • Content of the article does not answer any of the review questions, which was unclear during selection step 1 • No quantitative data could be retrieved from the article
Step 3. Screening during data-extraction phase
Further scrutiny of the article during the data-extraction phase possibly leading to exclusion

A manual search of reference lists of included articles and narrative reviews was also performed to find additional peer-reviewed literature which was not retrieved from PubMed or the Cochrane library.

### Gray literature and other data sources

An extensive search in other data sources was performed to retrieve information on epidemiological surveillance. Websites including WHO,^2,19^ FluNet,^36^ EuroFlu,^37^ European Centre of Disease Prevention and Control (ECDC),^38^ MoH of the countries of interest and national surveillance databases for influenza were searched. Other national surveillance networks and annual reports were searched using *Google*.

We referred to the WHO website to check recommendations on the vaccine composition in the Northern Hemisphere.^19^ Information on proportions of influenza B lineage circulation and circulation patterns for the 9 European countries selected for the analysis were obtained from the WHO-FluNet database, a global tool for influenza virological surveillance. Data are provided to this database remotely by National Influenza Centres (NICs) of the GISRS and other national influenza reference laboratories collaborating actively with GISRS, or are obtained from WHO regional databases.^36^ The EuroFlu website was reviewed to obtain influenza-related epidemiological and virological data for the selected countries in the European region. In each of the countries, one or several networks of sentinel physicians which report consultation rates due to influenza-like illness (ILI) and acute respiratory infection were reviewed.

All data reported are descriptive. Epidemiological parameters extracted from publications were incidence rates and proportions of influenza B among laboratory-confirmed influenza cases, with subtyping results. Outcome measures of disease burden included ER and GP visits, hospitalization and mortality rates, rates of complications and antibiotic consumption. Circulation patterns and vaccine mismatch were analyzed based on comparisons of vaccine and circulating B strains for each country by influenza season. Data on case distribution by influenza virus types and subtypes (B/Yamagata and B/Victoria), and age group are reported, where available.

## Abbreviations

*CI*: confidence interval
*ECDC*: European Centre of Disease Prevention and Control
*ER*: emergency room
*GISRS*: Global Influenza Surveillance and Response System
*GP*: general practitioner
*ILI*: influenza-like illness
*MoH*: Ministry of Health
*NICs*: National Influenza Centres
*WHO*: World Health Organization.
